# Genetic Analysis Workshop 16: Strategies for genome-wide association study analyses

**DOI:** 10.1186/1753-6561-3-s7-s1

**Published:** 2009-12-15

**Authors:** L Adrienne Cupples, Joseph Beyene, Heike Bickeböller, E Warwick Daw, M Daniele Fallin, W James Gauderman, Saurabh Ghosh, Ellen L Goode, Elizabeth R Hauser, Anthony Hinrichs, Jack W Kent, Lisa J Martin, Maria Martinez, Rosalind J Neuman, Michael Province, Silke Szymczak, Marsha A Wilcox, Andreas Ziegler, Jean W MacCluer, Laura Almasy

**Affiliations:** 1 Department of Biostatistics, Boston University School of Public Health, 801 Massachusetts Avenue, Boston, MA 02130 and Framingham Heart Study, Framingham, Massachusetts, USA; 2 Research Institute of the Hospital for Sick Children and University of Toronto, 555 University Avenue, Toronto, Ontario M5G 1X8, Canada; 3 Department of Genetic Epidemiology, University Medical Center Göttingen, Humboldtallee 32, 37073 Göttingen, Germany; 4 Division of Statistical Genomics, Washington University School of Medicine, 4444 Forest Park Boulevard, Campus Box 8506, St. Louis, Missouri 63108, USA; 5 Department of Epidemiology, Johns Hopkins Bloomberg School of Public Health, 615 North Wolfe Street, Baltimore, Maryland 21205, USA; 6 University of Southern California, Department of Preventive Medicine, Division of Biostatistics, 1540 Alcazar Street, CHP-220, Los Angeles, California 90033, USA; 7 Human Genetics Unit, Indian Statistical Institute, Kolkata 700018, India; 8 Department of Health Sciences Research, Mayo Clinic, 200 First Street Southwest, Rochester, Minnesota 55905, USA; 9 Duke University, Durham, North Carolina 27710 USA; 10 Department of Genetics, Southwest Foundation for Biomedical Research, P.O. Box 760549, San Antonio, Texas 78245, USA; 11 Division of Biostatistics and Epidemiology, Cincinnati Children's Hospital Medical Center, 3333 Burnet Avenue, Mail Code 5041, Cincinnati, Ohio 45229, USA; 12 INSERM, U.563, University Paul-Sabatier, CPTP, Toulouse F-31300, France; 13 Institut für Medizinische Biometrie und Statistik, Universität zu Lübeck, Universitätsklinikum Schleswig-Holstein, Campus Lübeck, Maria-Goeppert-Strasse 1, 23562 Lübeck, Germany; 14 Johnson & Johnson Pharmaceutical Research and Development, 1125 Trenton-Harbourton Road, Titusville, New Jersey 08560, USA

## Introduction

This supplement to *BMC Proceedings *contains the proceedings of Genetic Analysis Workshop (GAW) 16, which was held September 17-20, 2008, in St. Louis, Missouri, USA. Initiated in 1982, the GAWs are now held in even-numbered years with the purpose of evaluating strategies for detecting genetic effects of complex diseases, thought to be the result of the joint effects of environmental and genetic factors. Each GAW meeting begins with the distribution of datasets that those who attend the Workshop use for the purpose of developing and/or evaluating statistical methods. These datasets are jointly chosen for the next Workshop through a discussion of those attending the meeting and the GAW Advisory Committee. At most Workshops, GAW has included a set of simulated datasets, so that researchers can examine the behavior of statistical methods when knowing the answer. A primary goal of the Workshops is to focus discussion on specific topics of interest and areas of methodological concern. The datasets are generally available to any researcher who requests them. Each person who desires to attend the Workshops must participate in the evaluation of at least one of the distributed datasets, investigating novel approaches or comparing emerging and existing methods. Participants also include those who have provided the data or participate in the Workshop organization. More information about GAW, including details of upcoming Workshops, may be found at http://www.gaworkshop.org.

## Genetic Analysis Workshop 16

Genetic Analysis Workshop 16 focused its efforts on the evaluation of genome-wide association studies of large genomic chip datasets containing hundreds of thousands genotypes from single-nucleotide polymorphisms (SNPs). There were three problem datasets, two consisting of data from ongoing studies and one simulated. All three datasets consisted of phenotypic and genome-wide SNP scan data. Problem 1 data came from studies of rheumatoid arthritis (RA), Problem 2 included genotypic and phenotypic data from the Framingham Heart Study (FHS), and Problem 3 consisted of simulated phenotypic data using the pedigrees and genotypic data provided to GAW16 by the Framingham Heart Study. Each of these datasets is described in more detail in Amos et al. [1], Cupples et al. [2], and Kraja et al. [3]. Data for Problems 2 and 3 required an application to the database for Genotypes and Phenotypes (dbGaP) at the National Center for Biotechnology Information [4], which processed applications through the National Heart, Lung and Blood Institute, and distributed the data. To apply, researchers needed to have an eRA Commons account, to obtain Institutional Review Board approval, to ensure security of the data and to sign a data, distribution agreement in conjunction with an institutional signing official.

### Problem set 1

Data for Problem 1 was derived from a genome-wide study of RA. SNP genotype data were provided for 868 cases and 1,194 controls that had been assayed using an Illumina 550 k platform. The cases were independent individuals who had met the American College of Rheumatology criteria for RA. Four hundred forty-five cases came from a single member of sibling sets that were studied as a part of the North American Rheumatoid Arthritic Consortium (NARAC) because they had at least one additional sibling with rheumatoid arthritis; an additional 423 independent cases were included and were not selected for family history. The cases were recruited from across the United States and are predominantly of Northern European origin. The controls, derived from the New York Cancer Project, were enrolled in the New York metropolitan area and are somewhat enriched for individuals of Southern European or Ashkenazi Jewish ancestry compared with cases. Phenotypic data were also provided for *DRB1 *alleles, which were classified according to the RA shared epitope, levels of anti-cyclic citrullinated peptide, and levels of rheumatoid factor IgM.

### Problem set 2

Data for Problem 2 derived from a genome-wide scan conducted in Framingham Heart Study participants through the SNP Health Association Resource (SHARe). More detail describing this effort is included at the dbGaP [5]. Genotype data collected using Affymetrix 500 k (250 k Nsp and 250 k Sty) and 50 k gene centric platforms were provided for 6,848 participants with 6,621 in 766 pedigrees of three generations and 227 unrelated individuals. Phenotypic data for 7,130 participants were available for the first four examinations from the Original Cohort (recruited from 1948 to 1952) and Offspring Cohort (recruited from 1971 to 1975) and one examination for the Generation 3 Cohort (recruited from 2002 to 2005). These examinations were chosen because participants were approximately the same adult ages. Data included were demographics (sex and age), height, weight, and traditional risk factors for coronary heart disease (blood pressure and hypertension, diabetes and blood glucose, smoking, alcohol, and lipid levels). Additional data included, when appropriate, were age at onset of coronary heart disease, age at onset of diabetes, age at death, and age at last contact.

### Problem set 3

Phenotypic data for Problem 3 were simulated, using the pedigrees and genotypes from Problem 2. The simulated data were derived from a model emulating lipid traits and their relationships to cardiovascular disease. Two hundred simulated replicates were provided for GAW16. For each replicate there were 6,476 subjects in families from the FHS, with their actual genotypes for Affymetrix 550 k SNPs and simulated phenotypes. The total number of subjects and pedigree structures differed from those in Problem 2, because between the times that simulation began and data were made available, additional FHS participants provided consent for use of their data. Simulated phenotypes at three visits, 10 years apart, were generated for Problem 3. Up to six "major" genes influencing variation in high- and low-density lipoprotein cholesterol (HDL, LDL), and triglycerides (TG), and 1,000 "polygenes" were simulated for each trait. All polygenes act independently and have additive effects. A group of 39 polygenes influencing HDL were clustered on chromosome 11; otherwise, the polygenes for each trait were randomly distributed throughout the genome. At each simulated visit, individuals in the upper tail of the LDL distribution were designated as medicated. The proportion of subjects that are medicated increased across visits at 2%, 5%, and 15%. Coronary artery calcification (CAC) was simulated using age, lipid levels, and CAC-specific polymorphisms. The risk of myocardial infarction before each visit was determined by CAC and its interactions with smoking and two genetic loci. Smoking was simulated to be commensurate with rates reported by the Centers for Disease Control. The full model for these simulated data is included in Kraja et al. [3].

Individuals on the GAW mailing list of nearly 2,600 were notified through e-mail in Spring 2008 that data for the three Problems were available. A total of 183 groups requested GAW16 data: 124 for Problem 1 data and 59 for Problems 2 and 3 data, which needed to be accessed through dbGaP. In Summer 2008, 168 contributed papers were received describing analyses of these data sets. A book and CD containing these contributions plus descriptions of the data sets were distributed to GAW16 participants before the meeting in September.

The GAW16 participants included 240 individuals from all over the world, including Austria, Brazil, Canada, France, Germany, India, Korea, the Netherlands, Singapore, Spain, Taiwan, the United Kingdom, and the United States. The 168 contributions submitted to GAW16 were organized into 17 presentation groups of 7 to 18 papers each. These presentation groups were organized around the following themes: genome-wide association (GWA) for discrete traits; GWA for quantitative traits; multi-stage GWA strategies; haplotype-based analyses; controlling false-positive rates; multi-phenotype analyses; phenotype definition and development; quality control in GWA studies; machine learning; gene-gene interaction; gene-environment interaction; using gene expression, function, and pathways in GWA; combining information from linkage and association analyses; population and evolutionary genetics, including linkage disequilibrium patterns and population stratification; GWA analysis of longitudinal data; family-based GWA analyses; and gene- or region-based association analyses. Each presentation group was led by a person with previous GAW experience who facilitated group discussion, organized the group's oral presentation for the general GAW meeting, and took a lead in writing the group summary paper, which are published simultaneously with these proceedings in *Genetic Epidemiology *[6].

Members of presentation groups began interacting by e-mail and/or conference calls before GAW16, comparing and contrasting their approaches and results. Each presentation group met a full day at the Workshop, a first for GAW. During these meetings, they continued their discussions and finalized a group presentation, which was delivered to the full GAW16 audience during the general sessions on the subsequent two days. The group meetings were attended mostly by group participants, but were open to all GAW16 attendees. Seventy-two participants also contributed to poster sessions held during the general sessions. There also was a special general session on Novel Methods. Four papers submitted to GAW16 were selected before the meeting for presentation in this session because they had used or developed novel analytical approaches.

The 131 GAW contributions included in this issue of *BMC Proceedings *are a subset of the 168 contributions presented at GAW16. All contributions were peer-reviewed and selected on the basis of scientific merit.

The first three papers of these Proceedings describe the datasets. These are followed by the 131 individual GAW16 contributions organized by presentation group, and alphabetically by first author within each group. Additionally, in a supplement to the journal *Genetic Epidemiology*, published simultaneously with these Proceedings, a paper by each presentation group summarizes the contributions to that group and the lessons learned, comparing and contrasting contributions and describing their main themes and results. Overall, GAW16 generated many interesting discussions and some conclusions concerning appropriate approaches to the analysis of genome-wide association data. These discussions also highlighted areas in which further methodological development is needed.

## Competing interests

The authors declare that they have no competing interests.
